# Evolutionary shifts in the thermal biology of a subterranean mammal: the effect of habitat aridity

**DOI:** 10.1242/jeb.247048

**Published:** 2024-12-20

**Authors:** Hana N. Merchant, Daniel W. Hart, Nigel C. Bennett, Andries K. Janse van Vuuren, Marc T. Freeman, Andrew E. McKechnie, Chris G. Faulkes, Nathan D. Mordaunt, Steven J. Portugal

**Affiliations:** ^1^ Department of Biological Sciences, School of Life and Environmental Sciences, Royal Holloway University of London, Egham, Surrey TW20 0EX, UK; ^2^ School of Biological and Behavioural Sciences, Queen Mary University of London, London E1 4DQ, UK; ^3^ Department of Zoology and Entomology, University of Pretoria, Pretoria, Gauteng 0002, South Africa; ^4^ Mammal Research Institute, Department of Zoology and Entomology, University of Pretoria, Pretoria, Gauteng 0002, South Africa; ^5^ Department of Biology, University of Oxford, Oxford OX1 3SZ, UK

**Keywords:** Arid, Climate change, Environmental adaptation, Mammals, Temperature, Thermoregulation

## Abstract

Subterranean mammals representing a single subspecies occurring along an aridity gradient provide an appropriate model for investigating adaptive variation in thermal physiology with varying levels of precipitation and air temperature. This study examined the thermal physiological adaptations of common mole-rats (*Cryptomys hottentotus hottentotus*) across five populations along an aridity gradient, challenging the expectation that increased aridity would lead to reduced metabolic rate, lower body temperatures and broader thermoneutral zones. No significant, consistent differences in metabolic rate, body temperature or thermal conductance were observed between populations, suggesting uniform thermoregulatory mechanisms across habitats. Instead, behavioural strategies such as huddling and torpor may play a more prominent role than physiological adaptations in managing temperature regulation and water balance. The study also observed osmoregulatory differences, with populations employing distinct behavioural cooling strategies in response to water availability. These results underscore the need for further research into the responses of subterranean species to climate change, particularly in understanding how increasing global temperatures and aridification might influence species distribution if they lack the physiological capacity to adapt to future climatic conditions.

## Teaser

**Summary:** The thermal biology of *Cryptomys hottentotus hottentotus* does not vary along an aridity gradient. Instead, osmoregulatory traits appear to provide the basis for extreme arid adaptation, with implications for conservation of subterranean species under future climate scenarios.

## INTRODUCTION

Local adaptation is a key driver of phenotypic variation, enabling organisms to respond to diverse abiotic and biotic environmental conditions, such as fluctuations in ambient temperature (*T*_air_), precipitation and aridity. Phenotypic variation therefore allows species to inhabit a wide range of ecological niches (Bolte et al., 2009; Fox et al., 2019). Thermal physiological traits, particularly metabolic rate (MR), are known to shift in response to changes in *T*_air_ and aridity (Norin and Metcalfe, 2019). Endotherms, for instance, rely on resting metabolic energy for thermogenesis and heat dissipation to regulate core body temperature (*T*_b_) (Hayward et al., 2022). However, maintaining a constant *T*_b_ is energetically demanding, especially in resource-limited environments such as arid regions (Hayward et al., 2022).

The thermoneutral zone (TNZ) plays a crucial role in species survival across diverse environments. Defined as the range of *T*_air_ over which metabolic energy expenditure is minimised, the TNZ allows for thermoregulation without the need for additional heat production or dissipation (Kingma et al., 2014). When individuals experience *T*_air_ outside their TNZ, metabolic adjustments are required to either increase or decrease heat production, thereby preventing hyperthermia or hypothermia (Kingma et al., 2012). Metabolic rate and TNZ measurements are useful for assessing local adaptations to environmental factors such as *T*_air_ and aridity, revealing physiological variation even within a single species (Norin and Metcalfe, 2019; Merchant et al., 2024).

African mole-rats (Bathyergidae) are excellent models for studying local adaptation. Despite living in subterranean environments with relatively stable daily and seasonal temperatures (Bennett and Jarvis, 1988), mole-rats experience the effects of aboveground environmental conditions, such as food availability and soil composition (Šumbera et al., 2007). For instance, common mole-rats (*Cryptomys hottentotus hottentotus*) inhabit diverse habitats ranging from mesic to arid environments in western South Africa (Spinks et al., 2000). This makes common mole-rats an ideal candidate species for comparing physiological adaptations across different populations exposed to varying environmental conditions. Although convergent physiological adaptations to subterranean living are well documented in African mole-rats (Ivy et al., 2020; Logan et al., 2020; Hart et al., 2023; Faulkes et al., 2024), physiological differences attributed to environmental factors such as aridity and *T*_air_ are not yet fully understood (Wallace et al., 2021).

Several studies have explored the effects of micro- and macro-climates on the thermal physiology of *Cryptomys* subspecies. For instance, Mahali mole-rats (*Cryptomys hottentotus mahali*) inhabiting arid regions exhibit a broader TNZ compared with the highveld mole-rats (*Cryptomys hottentotus pretoriae*), indicating an ability to minimise energy expenditure over a wider range of *T*_air_ (Wallace et al., 2021). However, evaporative water loss (EWL), evaporative heat loss (EHL), and thermal conductance remained unchanged between the subspecies at higher *T*_air_, suggesting that macro-climates influence thermal physiology below the upper thermal critical limit and the micro-climate influences thermal physiology above the upper thermal critical limit (Wallace et al., 2021). Additionally, McGowan et al. (2020) examined the thermal responses of different mole-rat species to arid and mesic environments, highlighting non-metabolic mechanisms such as heat dissipation via ventral surfaces, used by some species to augment thermoregulation.

This study aimed to investigate resting metabolic rates (RMRs), *T*_b_, EWL rates, TNZs, thermal conductance and the EHL/MHP ratio in five populations of *C. h. hottentotus* along an aridity gradient. By holding individuals from these populations in identical artificial conditions, we sought to explore how differences in environmental conditions influence their thermal physiology. Based on the increased thermal and energy demands associated with arid regions, particularly foraging in dry soils (Grenfell and De Wall., 2024; Hart et al., 2021, 2022), we hypothesised that arid-dwelling populations would exhibit lower *T*_b_, RMR and EWL, along with broader TNZs, compared with mesic-dwelling populations (Wallace et al., 2021).

## MATERIALS AND METHODS

### Climate data

The Aridity Index (AI) is a numerical indicator of the degree of dryness of the climate at a given location (UNEP, 1992). The AI for the study populations was calculated from climate data (ranging from the years 1981–2020) retrieved from the ERA5-Land of the European Centre for Medium-Range Weather Forecasts, created by the Copernicus Climate Change Service (Muñoz-Sabater et al., 2021) with a spatial resolution of 0.1×0.1°. Monthly averaged temperature (*T*_a_ in °C), total precipitation (*P*_tot_ in m) and dew point temperature 2 m above the ground (*d*_2m_ in °C) were used. These data were used to calculate the annual AI (Eqn 1):
(1)

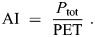
*P*_tot_ was directly obtained from ERA5-Land, and potential evapotranspiration (PET) was calculated from the Romanenko estimation (Eqn 2) (Romanenko, 1961):
(2)

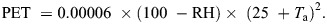


For Eqn 2, relative humidity (RH) was calculated from ERA5-Land *d*_2m_ (Eqn 3):
(3)

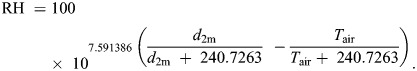


Aridity classifications and corresponding AI values, as outlined by UNESCO (1979) and UNEP (1992), state that where PET is greater than *P*_tot_, the climate is considered to be arid (Colantoni et al., 2015). The five sites used in this study are located along an aridity gradient and, based on the AI values at each of the five sites, range from arid to mesic: Steinkopf (AI=0.04, arid), No Heep (AI=0.07, arid), Klawer (AI=0.11, semi-arid), Darling (AI=0.42, mesic) and Somerset West (AI=0.86, mesic).

Surface precipitation and temperature were extracted for each population (Fig. S1). Soil temperature and volumetric soil moisture content were extracted at depths of 0–7 cm and 7–28 cm below the surface directly from ERA5-Land data (Fig. S1). These depths were chosen based on the average tunnel depth encountered at each of the five sites where common mole-rats were trapped, as outlined under ‘Animal capture’, below.

### Ethics and permitting

A collecting permit was obtained from the relevant nature conservation authorities (permit no. FAUNA 0419/2021, FAUNA 042/2021, CPB6-1161, CPB6-1163, CNN44-87-17699). Permission to capture common mole-rats was obtained from all landowners. The Animal Ethics Committee of the University of Pretoria evaluated and approved the experimental protocol (ethics clearance no. NAS016/2021) and DAFF section 20 approval was granted (SDAH-Epi-21031811071).

### Animal capture

Common mole-rats (*Cryptomys hottentotus hottentotus* Ellerman 1940) (*N*=60, 12 per population) were collected from five sites in South Africa, Steinkopf (29.261°S, 17.734°E; Northern Cape), No Heep (30.043°S, 17.959°E; Western Cape), Klawer (31.701°S, 18.745°E; Northern Cape), Darling (33.376 °S, 18.386°E; Western Cape) and Somerset West (34.076°S, 18.843°E; Western Cape). The presence of the study species at each site had previously been confirmed (Spinks et al., 2000; Visser et al., 2019; Hart et al., 2023). Sites were selected specifically to represent an aridity gradient, as outlined below.

All animals were captured during the non-breeding season (Spinks et al., 1999; Hart and Bennett, 2022) and only adult non-breeding colony members were used for this study. Breeding males were distinguishable from non-breeding males by their large descended abdominal testes and yellow staining around the mouth. Furthermore, the breeding males were usually, but not always, the largest male in each colony (Spinks et al., 2000; Hart et al., 2021). The breeding females were characterised by prominent axillary teats and a perforated vagina, which was absent in the non-breeding females. An equal number of males and females were used in this study.

Animals were transferred by road to the laboratory at the Department of Zoology and Entomology at the University of Pretoria, where they were housed in a climate-controlled room. The room regulated *T*_air_ at 23–25°C and a relative humidity of 40–60%. The light cycle of the room was set to 12 h light (06:00–18:00 h):12 h dark. Animals were housed for approximately 2 weeks prior to experiments, to allow individuals time to habituate after capture and fed *ad libitum*.

### Respirometry protocol

Two push-through respirometry systems were used simultaneously to measure rates of CO_2_ production (ml min^−1^) and H_2_O loss (g h^−1^), O_2_ consumption (ml min^−1^) and barometric pressure (kPa). Each system had a 3-l airtight custom-made Tupperware container attached with a fitted air inlet at the top and an outlet at the bottom, functioning as a metabolic chamber. A metal wire platform was custom-made to fit in the chamber, acting as a platform for the animal approximately 12 cm above the oil. At the bottom of each chamber was a layer of paraffin oil, approximately 1 cm deep, to eliminate evaporation produced by excreta. The chambers were placed in an incubator (Labcon low-temperature incubator, model L.T.I.E, South Africa) to control *T*_air_.

Air was pumped through tubing to a membrane drier (Champion^®^CMD3 air dryer and filter, Champion Pneumatic, Quincy, IL, USA), followed by a drying column containing Drierite^TM^ (anhydrous calcium sulfate; Hammond Drierite, Xenia, OH, USA) to scrub the air of moisture. Air was split into a baseline channel and an experiment channel that led to the chamber. A respirometry multiplex (MUX3-1101-18M, Sable Systems, Las Vegas, NV, USA) was used to sequentially sample by manually switching between baseline and experimental channels.

Subsampled air was pushed through an H_2_O analyser (Sable RH-200), O_2_ analyser (FC-10) and finally, a CO_2_ analyser (CA-10, all Sable Systems) measuring barometric pressure and CO_2_. The air was then vented via an outlet. Both channel flow rates were controlled by mass flow controllers (0-30 SLPM, Alicat Scientific Inc., Tucson, AZ, USA). Flow rates ranged from 3 to 6 l min^−1^ owing to daily fluctuations in air pressure. Tubing in the system was Bev-A-Line IV tubing (Thermoplastic Processes Inc., Warren, NJ, USA), used for all connections between chambers, analysers and the subsampler. All readings from analysers were digitised using a UI-2 analogue digital convertor (Sable Systems) and recorded with a sampling interval of 5 s using Expedata software (Sable Systems) on a laptop.

Core *T*_b_ was continuously measured using temperature-sensitive passive integrated transponder (PIT) tags (BioTherm, Biomark, Boise ID, USA) and a portable transceiver/data logger linked to an antenna (HPR+, Biomark) placed adjacent to the respirometry chamber. The PIT tags were injected intraperitoneally 3–7 days before starting experiments (Wallace et al., 2021). A 3-mm diameter thermistor probe (Sable Systems) was inserted through a hole, sealed with a rubber grommet, in the side of the metabolic chamber to measure *T*_air_ during gas exchange measurements.

### Respirometry experimental design

Individuals were fasted for 12 h before being placed in the respirometry chamber (Wallace et al., 2021). Fasting was undertaken to ensure individuals were in a post-absorptive state, in line with the requirements for measuring RMR (McNab, 1997). Prior to each experiment, the individual was weighed on an electronic balance (EJ-160, A&D, Tokyo, Japan) and the unique PIT tag number was logged, along with colony details, sex and breeding status. The animal was then placed in a metabolic chamber in the incubator at 10°C for 1 h to acclimatise. After this period elapsed, the experiment began with a baseline reading taken for 5 min. The individuals then were exposed to a stepped profile of increasing *T*_air_ from 10°C up to the *T*_air_ at which their *T*_b_ reached 42°C, based on previous studies on this species and similar to other small mammals (van Jaarsveld et al., 2021; Wallace et al., 2021). Each individual was measured at each *T*_air_ setpoint for 5 min before the samplers were switched from the experimental to the baseline channel while the incubator temperature was increased to reach the next *T*_air_ setpoint. Between 10 and 25°C, *T*_air_ was increased in 5°C increments. From 25°C onwards, the *T*_air_ was increased in 2°C increments. Once *T*_b_ reached 42°C, the experiment ended, and the animal was taken out of the chamber. The animal was then weighed again. The animal was returned to the colony or box at room temperature to rest, with *ad libitum* water and food.

Expedata traces for each individual were first corrected for O_2_ drift, and lag using the relevant algorithms in Expedata (Sable Systems). Values for each baseline interval and experimental reading at each *T*_air_ were extracted manually for CO_2_, O_2_, H_2_O, barometric pressure (BP in kP) and *T*_b_. Eqns 4 and 5 (Lighton, 2018) were used to calculate animal rates of CO_2_ and water vapour production (*V̇*_CO_2__ and *V̇*_H_2_O_), and EWL was calculated from the lowest 5-min steady-state trace for each reading, assuming 0.803 mg ml^−1^ H_2_O vapour:
(4)

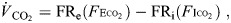

(5)

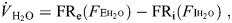


where FR_e_ is the volume of air expired, FR_i_ is the volume of air inspired, *F*e_CO_2__ is the expired CO_2_ fraction, *F*i_CO_2__ is the inspired CO_2_ fraction, *F*e_H_2_O_ is the volume of water expired and *F*i_H_2_O_ is the volume of water inspired. Resting metabolic rate (W) was calculated from *V̇*_CO_2__ using 27.8 J ml^−1^ CO_2_ (Walsberg and Wolf, 1995). Evaporative heat loss (EHL in W) was calculated from the assumed latent heat of evaporation of water of 2.406 J mg^−1^ H_2_O (Tracy et al., 2010). Heat dissipation capacity and evaporative cooling were calculated as evaporative heat loss/metabolic heat production (EHL/MHP). Dry conductance (*C*_dry_ in W m^−2^ °C^−1^) was calculated using Eqn 6:
(6)

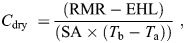
where SA is body surface area (cm^2^) predicted using Eqn 7 (Calder, 1996):
(7)

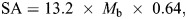
where *M*_b_ is body mass (g).

### Age estimation

Ageing was carried out on the extracted skulls for each of the 60 individuals (Merchant et al., 2024b). Four age classes were discerned from sequential tooth wear and eruption patterns of the upper molars in accordance with Bennett et al. (1990), with age class 1 being the youngest and 4 the oldest. The right side of the jaw was used in all individuals for consistency.

### Statistical analysis

All calculations and statistical analyses were performed using the statistical software R version 4.2.2 (https://www.r-project.org/). The inflection values, above which *T*_b_, MR, EWL, EHL/MHP and *C*_dry_ increased rapidly as a function of *T*_air_, and the lower critical limit of thermoneutrality were identified using the ‘segmented.lme’ package (Muggeo et al., 2014). Individual was included as a random effect. Several individuals, particularly those form the arid environments, were identified to be in torpor during the experimental procedure, and data from these individuals were excluded from all analyses. *T*_b_ was analysed above and below the inflection points. *C*_dry_ was analysed only below the inflection points, and EWL and EHL/MHP only above the inflection points. MR was analysed between the inflection points (within the TNZ). Finally, the slopes for the relationships of thermoregulatory response variables at different *T*_air_ values were analysed. Analyses were carried out using generalised linear models and mixed effects models in the package ‘nlme’. A generalised variance inflation factor (GVIF) was used to determine multicollinearity between predictor traits, to account for the mix of continuous and categorical traits, undertaken in a stepwise fashion. The results of the VIF meant that ‘colony’ was removed. The ‘dredge’ function from the package ‘MuMIn’ was used to carry out model selection. Initial models included *T*_air_, body mass, age and population. Days in captivity were also included in the models to determine the influence of the length of time between capture and the date of the experiment. Models with the highest rank were selected using AIC values (Grueber et al., 2011). Pseudo-replication was accounted for by including individual as a random factor in all analyses. Significance was determined at *P*<0.05.

Additionally, five ANOVAs were carried out for each of the thermoregulatory response variables (*T*_b_, MR, EWL and EHL/MHP) at five specific temperatures chosen to represent a range of temperatures experienced by the populations used in this study: 15°C (minimum *T*_air_ experienced by any of the populations), 27°C (*T*_air_ at which the lowest inflection point was found for any of the populations), 33°C (*T*_air_ at which every population is in their TNZ), 37°C (*T*_air_ at which the highest inflection point was found for any of the populations) and 25°C (highest *T*_air_ experienced by the populations). *C*_dry_ was only analysed at temperatures below the TNZ (15°C and 25°C). These *T*_air_ values were determined using the ERA5-Land dataset created by the Copernicus Climate Change Service (Muñoz-Sabater et al., 2021) for soil temperature at 7–28 cm below the surface, which was selected based on the depth of *C. h. hottentotus* tunnel systems (Spinks et al., 2000).

Two additional measurements were calculated for the breadth of the TNZ (TNZ range), determined by subtracting the lower inflection point from the upper inflection point. The second measurement, thermal range, was determined by the difference between the maximum and minimum *T*_air_ each individual experienced. Thermal range was calculated by subtracting the *T*_air_ of the first reading (lowest temperature value) from the final reading (highest temperature value).

Further ANOVAs were carried out to determine variation between the populations in TNZ range (Table S1) and thermal range (Table S2). Additionally, a preliminary ANOVA test for the effect of colony on each variable was carried out, with no differences identified between the different colonies within each population (Table S3).

## RESULTS

### Body temperature

**Fig. 1.**
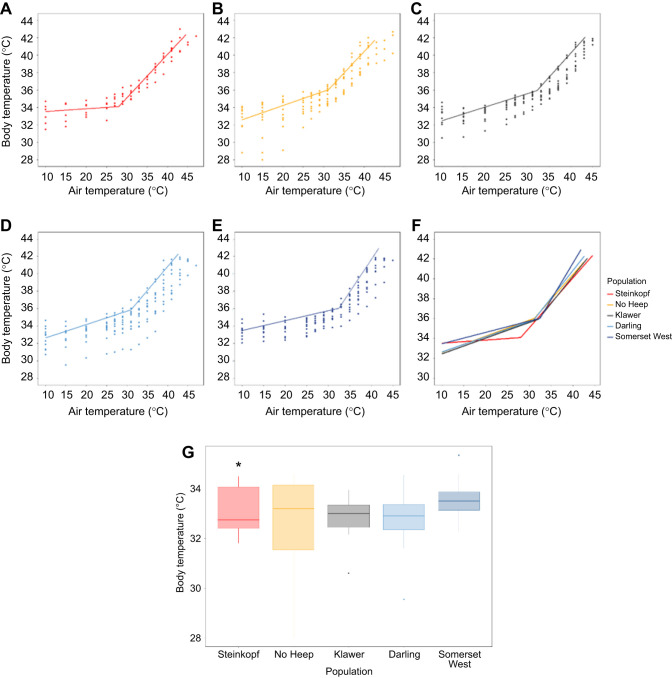
**Body temperature (*T*_b_) as a function of air temperature (*T*_air_) for *Cryptomys hottentotus hottentotus*.** Data are shown for each population individually from most arid to mesic: (A) Steinkopf, (B) No Heep, (C) Klawer, (D) Darling and (E) Somerset West. Each individual is shown (*N*=55), coloured by population. Outliers in C and D correspond to a single individual. Mean population values are shown in F, with each population colour-coded. Regression lines are based on the segmented linear models used to identify inflection points. (G) Significant differences in *T*_b_ at 15°C are shown for each population of *C. h. hottentotus*. Asterisk indicates the population that showed significant differences to other populations.

Mean *T*_b_ values for each population were: Steinkopf=36.6°C, No Heep=35.8°C, Klawer=35.9°C, Darling=35.6°C, Somerset West=36°C. A linear mixed effects model (LMM) revealed that across all experimental *T*_air_ values, individuals from the No Heep population had significantly lower *T*_b_ values than individuals from Darling (LMM: *t*=2.04, *P*=0.04), whereas no other differences were found between the populations (Fig. 1A–F). ANOVA and *post hoc* Tukey tests showed that at *T*_air_=15°C, the *T*_b_ of animals from Steinkopf had a significantly lower *T*_b_ than the animals from Somerset West (ANOVA: *F*=1.003, d.f.=4, 47, *P*=0.04; Fig. 1G). There were no differences at any other air temperatures and days in captivity demonstrated no effect on body temperature.

### Resting metabolic rate

**Fig. 2.**
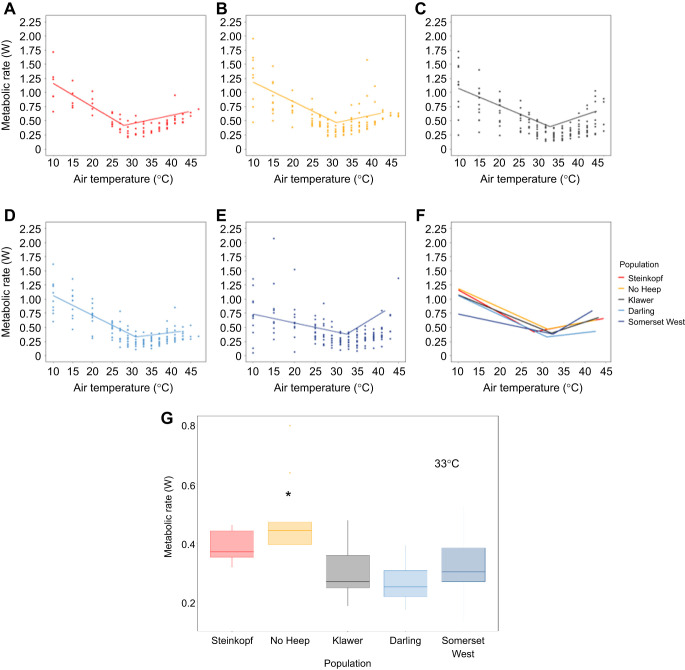
**Metabolic rate (MR, in W) against air temperature (*T*_air_) for *C.*** ***h. hottentotus*****.** Data are shown for each population individually from most arid to mesic: (A) Steinkopf, (B) No Heep, (C) Klawer, (D) Darling and (E) Somerset West. Each individual is shown (*N*=55), coloured by population. Mean population values are shown in F, with each population colour coded. (G) Significant differences in MR at 37°C for each population of *C. h. hottentotus*. Asterisk indicates the population that showed significant differences to other populations.

The thermoneutral RMR (i.e. MR within the TNZ) of individuals from No Heep was higher than all other populations (GLM: *F*=0.5254, d.f.=4,1,3,1, *P* <0.05) (Table 1). A LMM showed that, overall, the populations of *C. h. hottentotus* did not differ in MR over the range of experimental *T*_air_, and body mass was a significant predictor of MR (Fig. 2A–F). At *T*_air_=37°C, the animals from No Heep had a higher mean MR than the Darling (ANOVA: *F*=6.06, d.f.=4, 45, *P*<0.001), Somerset West (ANOVA: *F*=6.06, d.f.=4, 45, *P*=0.008) and Klawer (ANOVA: *F*=6.06, d.f.=4, 45, *P*=0.004) populations (Fig. 2G). There were no differences at any other experimental *T*_air_ values and days in captivity had no effect on metabolic rate.

**Table 1.**
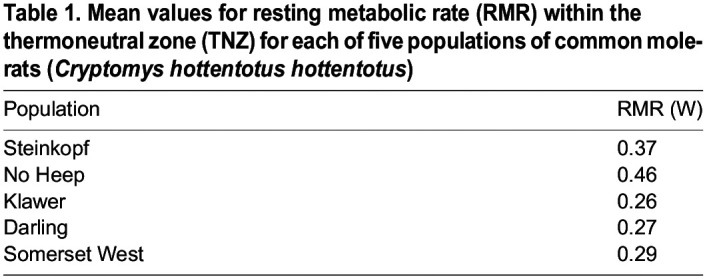
Mean values for resting metabolic rate (RMR) within the thermoneutral zone (TNZ) for each of five populations of common mole-rats (*Cryptomys hottentotus hottentotus*)

The slopes of MR revealed no differences between any of the populations below the TNZ (ANOVA: *F*=1.049, d.f.=4, 53, *P*=0.39), or above (ANOVA: *F*=1.709, d.f.=4, 53, *P*=0.16). The thermal range of the TNZ did not differ between populations, except for Steinkopf, where the thermal range was significantly higher than that of No Heep and Somerset West. The TNZ breadth of the mole-rats from the Somerset West population was significantly shorter than that of the Darling and Steinkopf mole-rat populations (Table 2).

**Table 2.**
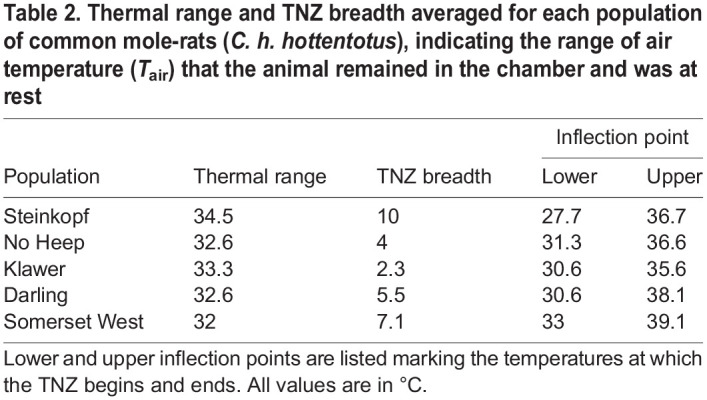
Thermal range and TNZ breadth averaged for each population of common mole-rats (*C. h. hottentotus*), indicating the range of air temperature (*T*_air_) that the animal remained in the chamber and was at rest

### Evaporative water loss

**Fig. 3.**
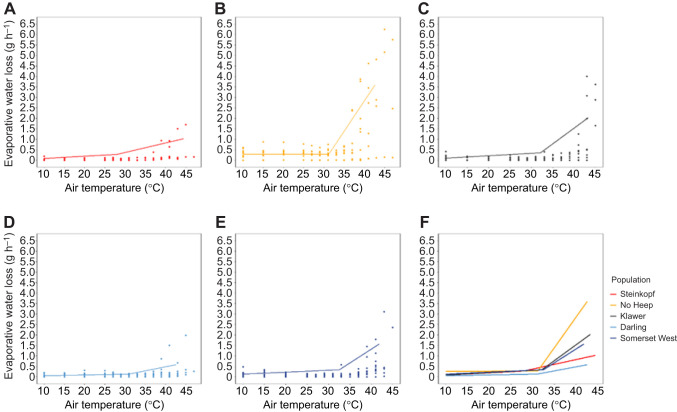
**Evaporative water loss (EWL) as a function of air temperature (*T*_air_) for *C.*** ***h. hottentotus*****.** Data are shown for each population individually from most arid to mesic: (A) Steinkopf, (B) No Heep, (C) Klawer, (D) Darling and (E) Somerset West. Each individual is shown (*N*=55), coloured by population. Mean population values are shown in F, with each population colour-coded.

**Fig. 4.**
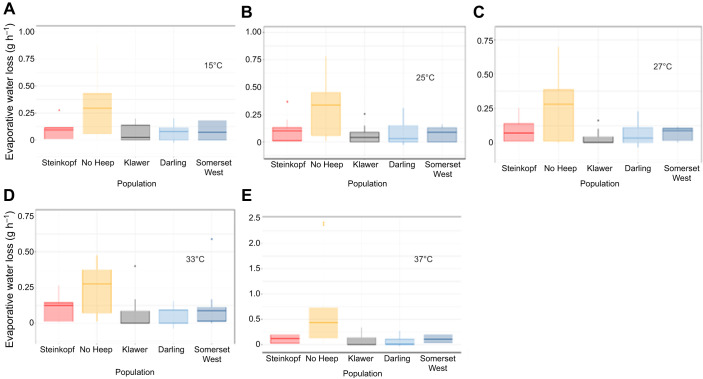
**Evaporative water loss (EWL) (g** **h^−1^) at different air temperatures for each population of *C. h. hottentotus*.** (A) 15°C, (B) 25°C, (C) 27°C, (D) 33°C and (E) 37°C.

The mole-rats at No Heep had higher EWL than all other populations above the inflection point (GLM: *F*=6.19, d.f.= 4, 1, 3, 1, *P*<0.05) and days in captivity was a significant predictor of EWL (GLM: *F*=2.98, d.f.=4, 1, 3, 1, *P*<0.05), with increased water loss in the two arid populations compared with the mesic and intermediate populations. Additionally, there was a significant difference in EWL between mole-rats from age classes 2 and 4. An LMM showed that body mass was a significant predictor (Fig. 3) of EWL, and body mass at No Heep was the only significant interaction. At all *T*_air_, the EWL in mole-rats from No Heep was higher than all other populations (Fig. 4).

### Evaporative heat loss/metabolic heat production

**Fig. 5.**
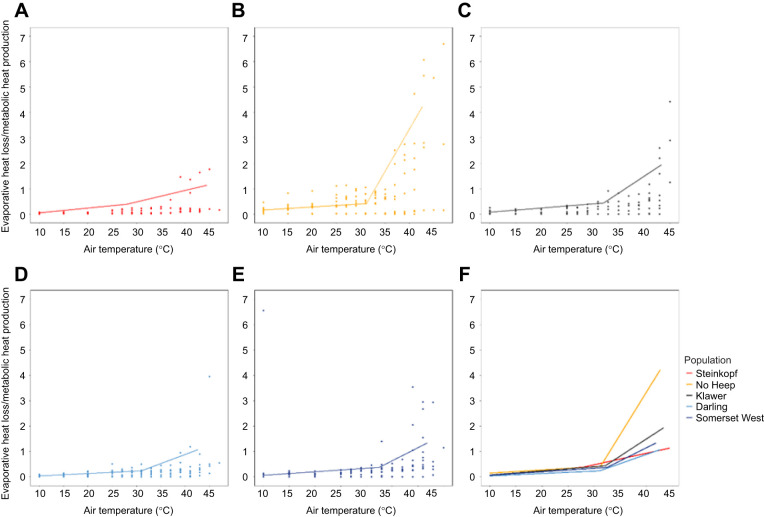
**Evaporative heat loss/metabolic heat production (EHL/MHP) against air temperature (*T*_air_) for each population of *C. h. hottentotus*.** Data are shown for each population individually from most arid to mesic: (A) Steinkopf, (B) No Heep, (C) Klawer, (D) Darling and (E) Somerset West. Each individual is shown (*N*=55), coloured by population. Mean population values are shown in F, with each population colour-coded.

**Fig. 6.**
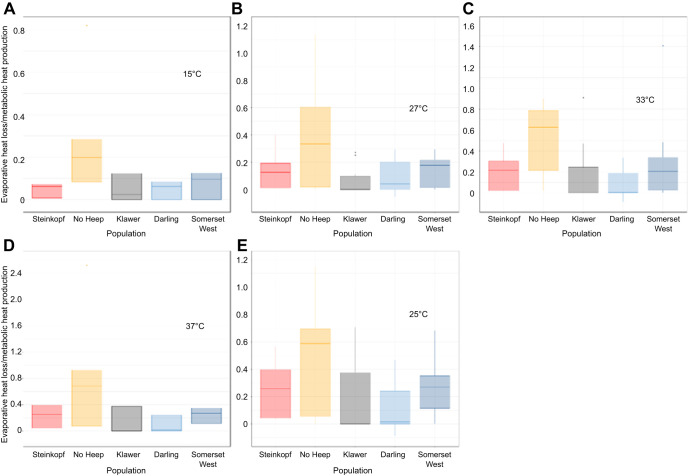
**Evaporative heat loss/metabolic heat production ratio at different air temperatures for each population of *C. h. hottentotus*.** (A) 15°C, (B) 27°C, (C) 33°C, (D) 37°C and (E) 25°C.

Individuals from No Heep had significantly higher EHL/MHP than individuals from Darling, Klawer and Steinkopf above the inflection point (GLM: *F*=3.1451, d.f.=4, 3, *P*<0.05). An LMM showed that body mass in the mole-rats from the No Heep population was a significant interaction, indicating that water loss varied more with body mass for No Heep individuals compared with other populations (Fig. 5). Significant differences in EHL/MHP were found between age classes 2 and 4 in the LMM. At all *T*_air_ values, the values from the population at No Heep were higher than all other populations (ANOVA: *F*=4.35, d.f.=4, 45, *P*<0.05) (Fig. 6).

### Conductance

**Fig. 7.**
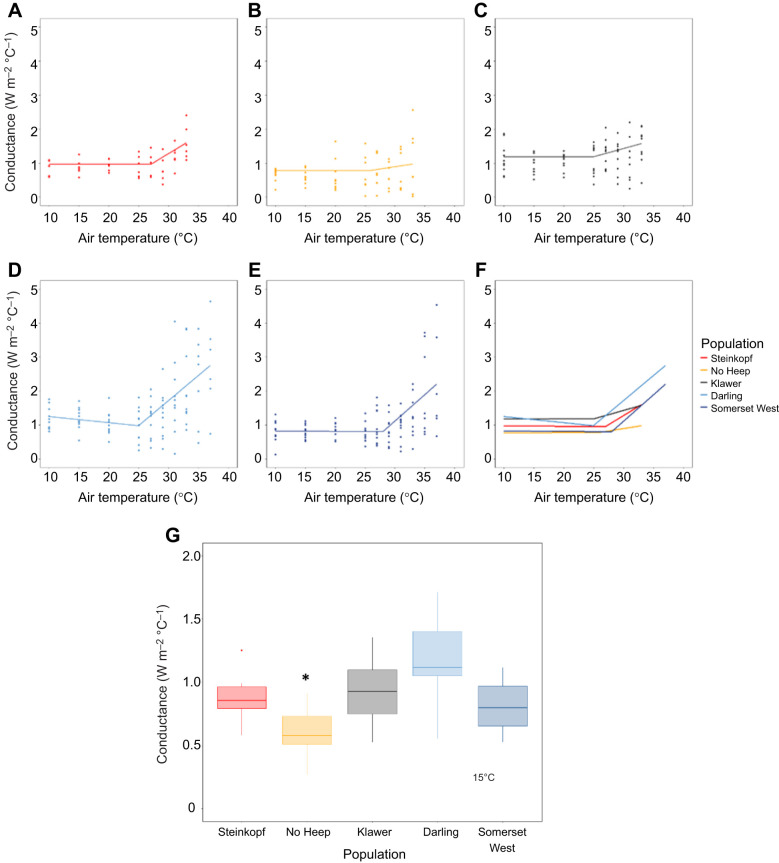
**Conductance (*C*_dry_, W** **m^−2^** **°C^−1^) against air temperature (*T*_air_) for *C.*** ***h. hottentotus***. Data are shown for each population individually from most arid to mesic: (A) Steinkopf, (B) No Heep, (C) Klawer, (D) Darling and (E) Somerset West. Each individual is shown (*N*=55), coloured by population. Mean population values are shown in F, with each population colour-coded. (G) Significant differences in *C*_dry_ at 15°C for each population of *C. h. hottentotus*. Asterisk indicates the population that showed significant differences to other populations.

Mean conductance (*C*_dry_) values for each population were: Steinkopf=0.9 W m^−2^ °C^−1^, No Heep=0.7 W m^−2^ °C^−1^, Klawer=1.1 W m^−2^ °C^−1^, Darling=1.2 W m^−2^ °C^−1^ and Somerset West=0.9 W m^−2^ °C^−1^. An LMM carried out on *C*_dry_ values below the TNZs for each population showed that there were no significant predictors for thermal conductance (Fig. 7). At 15°C, animals from No Heep had a lower thermal conductance than those animals from Darling (ANOVA: *F*=7.96, d.f.=4, 42, *P*<0.001) and Somerset West (ANOVA: *F*=7.96, d.f.=4, 42, *P*<0.001) (Fig. 7G). There were no differences in thermal conductance between the mole-rat populations at any other *T*_air_.

## DISCUSSION

The results of this study challenge prevailing assumptions about the thermal physiological adaptations of common mole-rats to arid environments. Contrary to the prediction that mole-rats in more arid habitats would exhibit reduced MR and *T*_b_, and broader TNZs, our findings show no significant, consistent variation in these parameters across populations distributed along an aridity gradient. Although some differences were observed, they lacked consistency, suggesting that thermal biology in common mole-rats may not respond as expected to local environmental conditions like aridity.

At the regional level, our findings indicate that MR and *T*_b_ do not differ among populations in response to variations in aridity. This aligns with studies on other *Cryptomys* subspecies, such as *C. h. mahali* and *C. h. pretoriae* (Wallace et al., 2021), where thermal conductance also did not vary with habitat. One potential explanation for these results is that mole-rats across all populations may utilise similar thermoregulatory mechanisms – whether metabolic or behavioural – suggesting a convergent adaptation to their relatively stable, subterranean microclimates.

The possibility that group size influences thermoregulation offers another perspective. According to the aridity food distribution hypothesis, social living may alleviate the need for physiological adaptations to cope with the increased energy demands associated with foraging for patchily distributed food in arid regions (Jarvis et al., 1994; Hart et al., 2022). Although theoretical models predict larger group sizes in arid regions (Spinks and Plaganyi, 1999), the evidence remains inconclusive (Spinks et al., 2000).

Social behaviors such as huddling may also play a significant role in *T*_b_, particularly in colder, arid environments, where heat loss can be more pronounced at low temperatures (Kotze et al., 2008). In this study, several individuals from the Steinkopf population – one of the arid populations – exhibited *T*_b_ values between 24 and 28°C at lower *T*_air_ values, indicating potential torpor use. This mirrors findings in *Cryptomys* subspecies such as Natal mole-rats (*C. h. natalensis*), where torpor is used as a physiological adaptation strategy (Oosthuizen et al., 2021; Finn, 2022). However, the role of huddling in these responses remains unclear, as our experiments measured individuals in isolation, a condition that might not reflect their natural social settings.

The lack of significant physiological differences across populations suggests that mole-rats may rely more on behavioural thermoregulation rather than metabolic flexibility to cope with varying environmental conditions. Similar findings have been reported in other subterranean rodents, such as coruros (*Spalacopus cyanus*), which show little change in basal metabolic rate or maximum metabolic rate when acclimated to different thermal environments (Nespolo et al., 2001). This may indicate that the microclimatic stability of burrow systems limits the need for metabolic plasticity.

### Water balance

Water availability in mole-rats is closely tied to food availability, as they derive most of their water from their diet (Bennett and Jarvis, 1995; Jarvis et al., 1998). Larger social groups may increase foraging efficiency, which could mitigate water stress in arid environments (Hart et al., 2022). Observations during our study support the idea that mole-rats employ various osmoregulatory strategies in response to arid conditions. For instance, individuals from mesic populations were more likely to use urine for evaporative cooling (urohydrosis) at higher *T*_air_ values, while individuals from arid populations did not exhibit this behaviour. Instead, they used panting, a water-efficient cooling mechanism well-suited to dry conditions (Šumbera, 2019).

Interestingly, individuals from the No Heep population – one of the arid populations – exhibited significantly higher EWL and evaporative cooling efficiency than the other populations. This could indicate physiological stress, potentially linked to food scarcity and shallow burrows that expose the population to more extreme surface temperatures. The shallow burrow systems observed in No Heep (5–10 cm deep) contrast with the deeper burrows (10–20 cm) observed in other populations, potentially leaving these mole-rats more vulnerable to environmental fluctuations. Further studies comparing this population with terrestrial mammals might offer insights into how burrow depth and water availability influence physiological responses to heat (Chalwin-Milton et al., 2024).

Days in captivity was found to influence EWL. As the number of days in captivity increased, the two arid populations showed an increase in water loss, then a decrease. The water loss differences in No Heep could be linked in part to the length of time in captivity, as the arid populations were potentially influenced by the ambient captive conditions owing to the greater degree of change compared with the natural conditions of the mesic populations. No other thermoregulatory trait demonstrated an influence of days in captivity, thus it is possible to say that overall the results from this study demonstrate that thermoregulation and heat dissipation reflect the natural conditions in which these individuals lived.

### Conclusions

This study provides evidence that common mole-rats do not rely on significant changes in physiological thermoregulatory mechanisms to cope with the arid conditions in which they live. Instead, osmoregulatory strategies, such as urohydrosis and panting, appear to be more important in managing heat dissipation at high *T*_air_ values. These findings suggest that the relatively stable microclimates of subterranean habitats buffer mole-rats from the more extreme surface conditions. However, individuals in certain populations, such as those from No Heep, may be more exposed to surface environmental conditions, leading to increased physiological stress.

As global temperatures continue to rise, the future of subterranean species such as mole-rats remains uncertain. Their role in ecosystems – such as in soil turnover and root control – makes understanding their thermal physiology and osmoregulatory strategies crucial. Given the limited knowledge of how subterranean species will cope with climate change, further research into the thermal biology and water-use strategies of these animals is essential. Understanding these mechanisms will not only inform conservation strategies but also provide insights into the broader impacts of climate change on ecosystems where subterranean species play a key role.

## Supplementary Material

10.1242/jexbio.247048_sup1 Supplementary information
