# Supplementary material for: Evolutionary shifts in the thermal biology of a subterranean mammal: the effect of habitat aridity
Source: J Exp Biol. 2024 Dec 20;227(24):jeb247048. doi: 10.1242/jeb.247048 (PMC11698034; doi:10.1242/jeb.247048)
Supplement: Supplementary information [file jexbio-227-247048-s1.pdf]

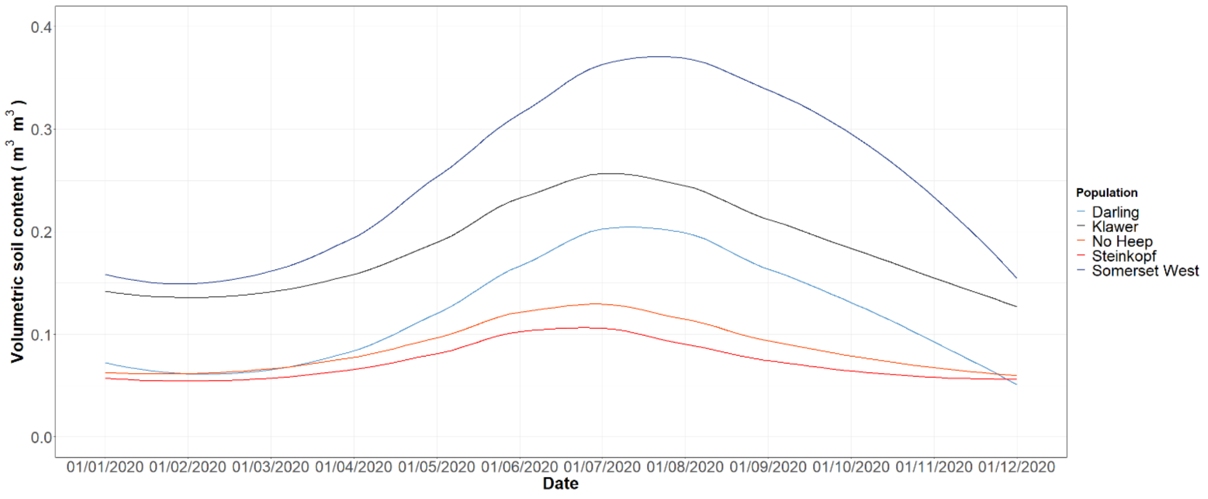

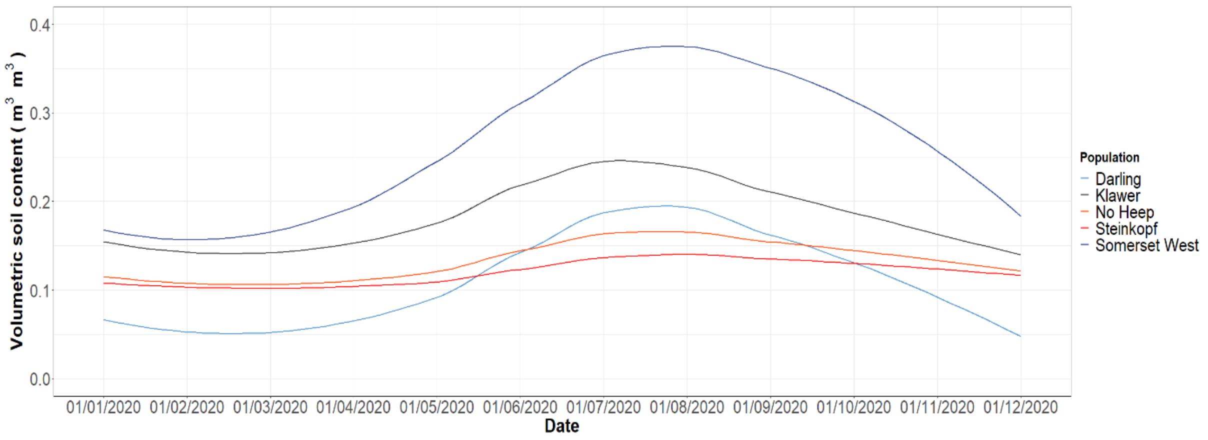

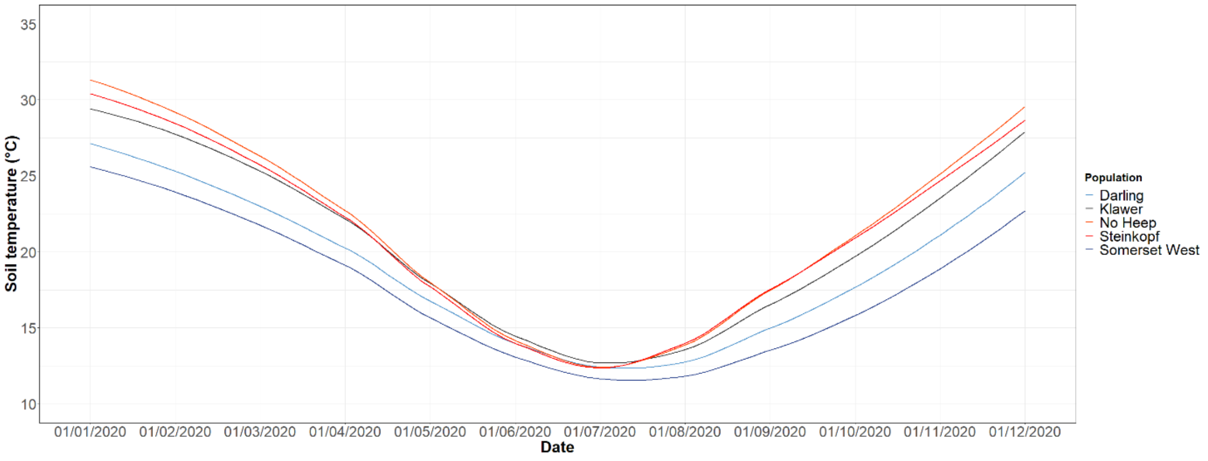

Journal of Experimental Biology • Supplementary information

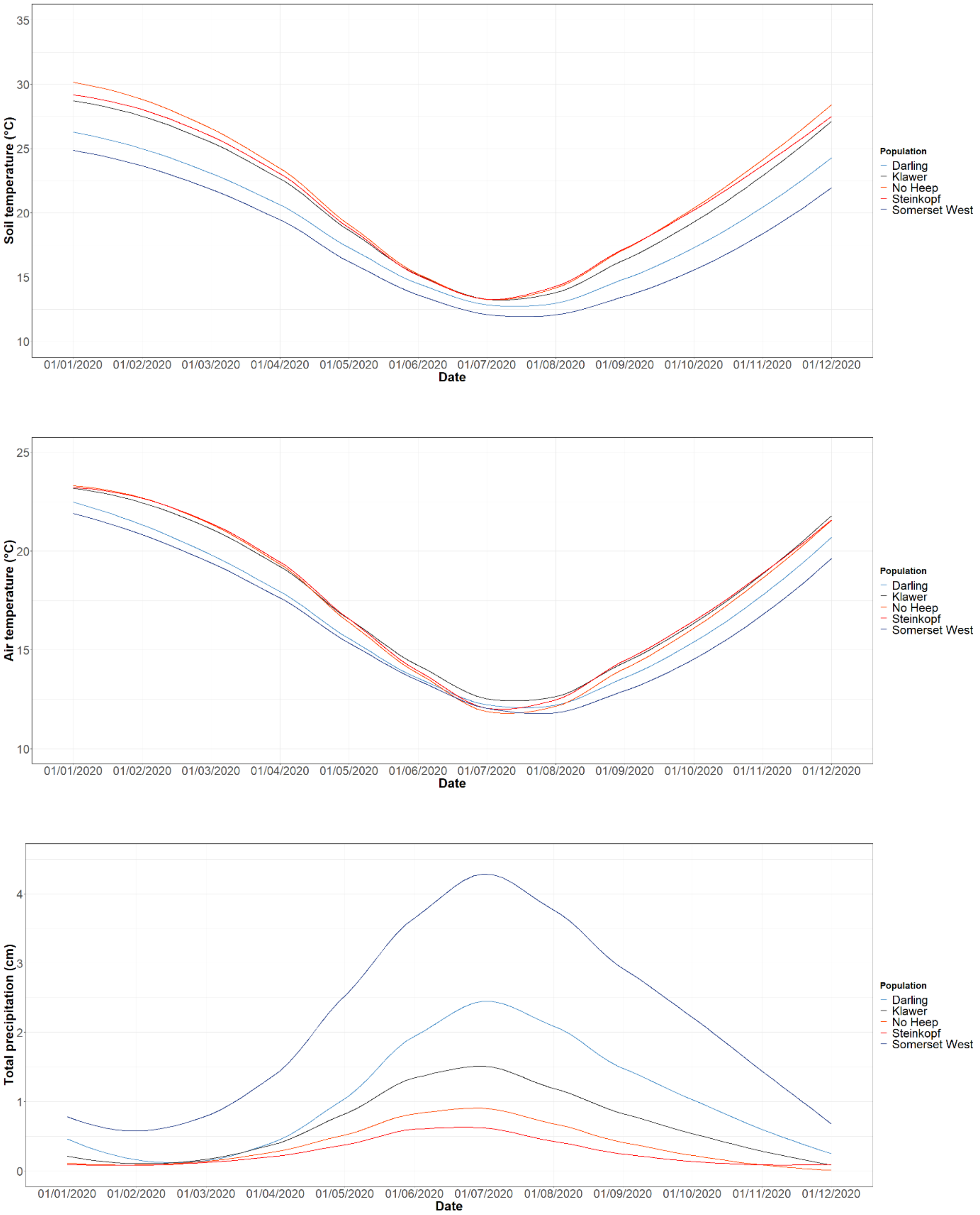

Fig. S1. Volumetric soil moisture content (m^3 m^{-3}) at 0-7cm (a), and 7-28cm (b) depth; soil temperature (°C) at 0-7cm (c) and 7-28cm (d) depth for each of the 5 populations for each month in the year 2020. Temperature of the soil at 2m above the soil surface (e) and accumulated liquid and frozen water that falls to the Earth's surface (f) at each of the 5 locations for every month in the year 2020. All data is based on climate data retrieved from ERA5-Land dataset.

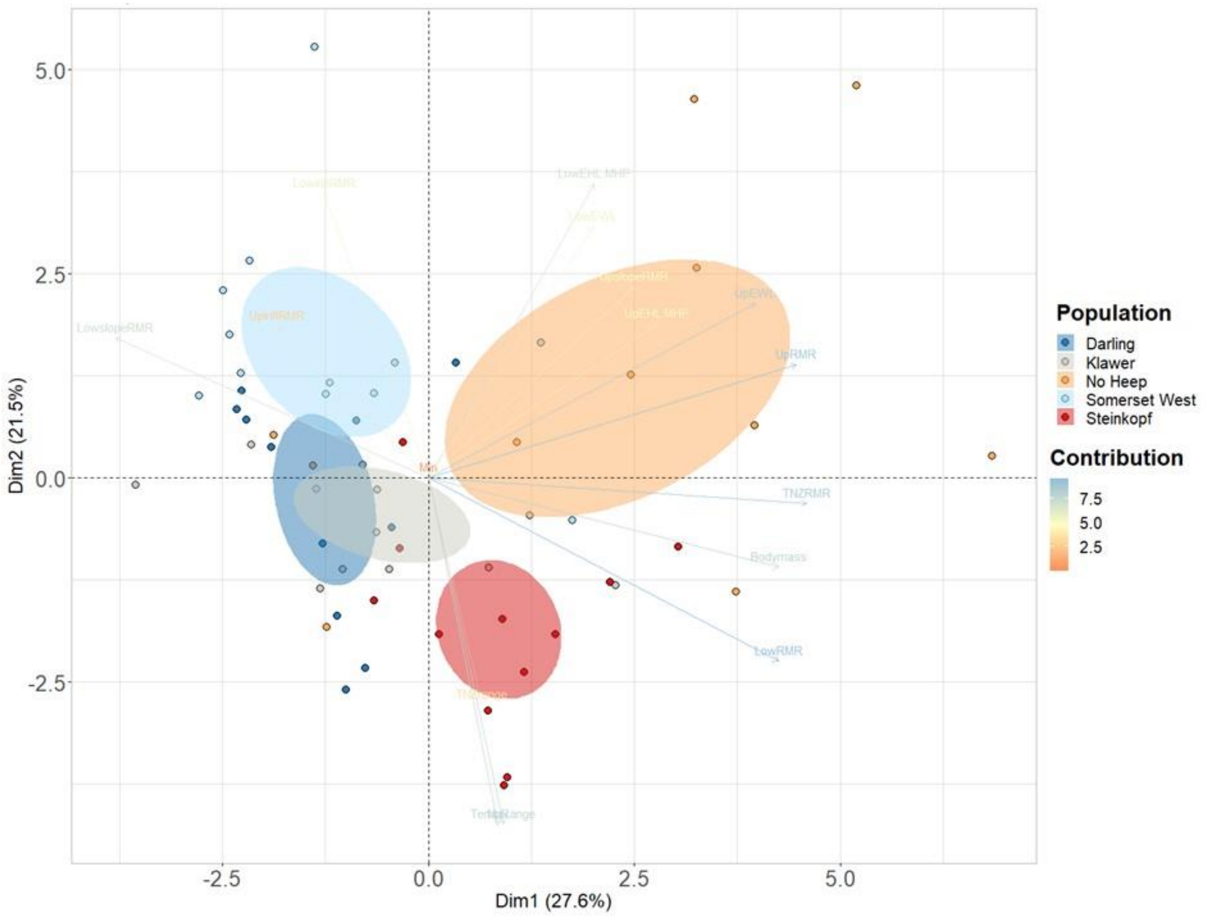

Fig. S2. Principal component analysis based on various measures of metabolic traits of 60 individuals of *C. h. hottentotus* across 5 populations, Darling, Somerset West, Klawer, No Heep and Steinkopf. The first (Dim1) and second (Dim2) principal component display 27.6% and 21.5% of the total variation, respectively. Confidence ellipses are shaded according to population colour and define the region containing 95% of samples in relation to the underlying Gaussian distribution. Contributions of each variable used in the PCA analysis are displayed using a gradient, blue indicating the highest contribution and red the lowest contribution.

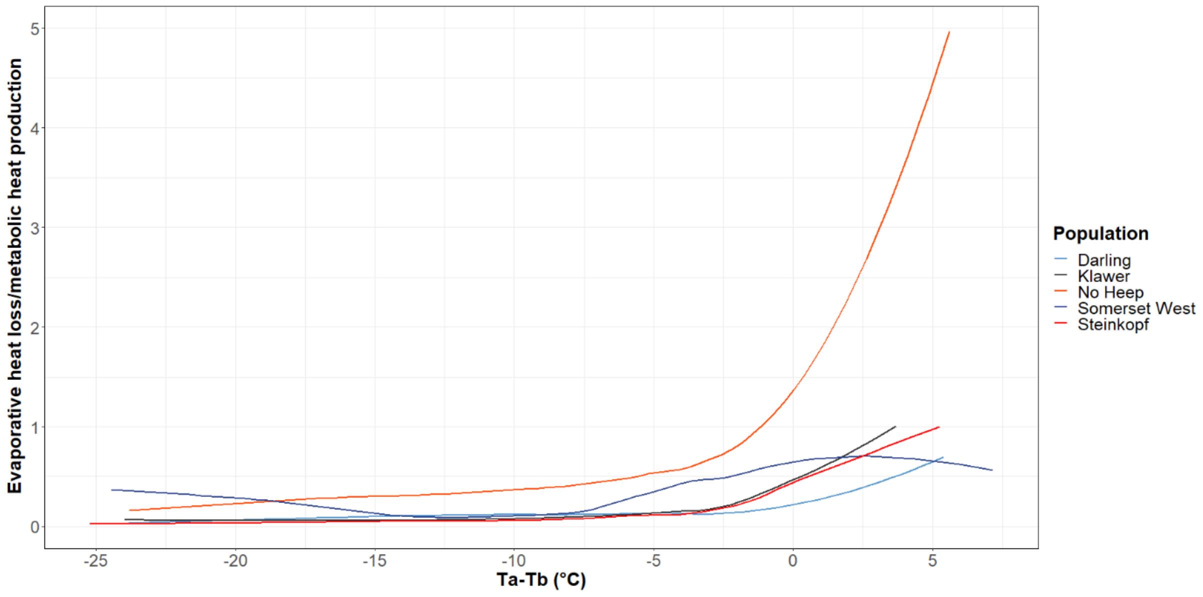

Fig. S3. Body temperature subtracted from ambient temperature (T_a-T_b) against evaporative heat loss/metabolic heat production (EHL/MHP) for each population of common mole-rats (*C. h. hottentotus*) at each temperature during which they were in the respirometry chamber.

Table S1. ANOVA results table of TNZ range between each of the 5 populations of common mole-rats (*C. h. hottentotus*). No significant difference was found between any of the populations.

	Df	Sum Sq	Mean Sq	F value	Pr(>F)
**Population**	4	66.64	16.66	3.8	0.09
**Residuals**	45	223.57	4.38		

Table S2. ANOVA results table of thermal range between each of the 5 populations of common mole-rats (*C. h. hottentotus*). No significant difference was found between any of the populations.

	Df	Sum Sq	Mean Sq	F value	Pr(>F)
**Population**	4	41.85	10.46	2.38	0.06
**Residuals**	45	237.88	4.4		

Journal of Experimental Biology • Supplementary information

Table S3. A series of ANOVA results tables to determine within population differences between each colony for each of the physiological measures: metabolic rate (MR) in the TNZ (TNZMR), evaporative water loss (EWL) above the inflection point (UpEWL); metabolic heat production (EHL/MHP) above the inflection point (UpEHL/MHP). No significant differences were found between colonies within the same population of common mole-rats (*C. h. hottentotus*). Any significance indicates differences between colonies from different populations.

TNZMR

	**Df**	**Sum Sq**	**Mean Sq**	**F value**	**Pr(>F)**
**Colony**	24	0.56	0.02	1.91	0.05
**Residuals**	30	0.37	0.01		

UpEWL

	**Df**	**Sum Sq**	**Mean Sq**	**F value**	**Pr(>F)**
**Colony**	24	27.56	1.15	5.39	p<0.01
**Residuals**	30	6.39	0.21		

UpEHL/MHP

	**Df**	**Sum Sq**	**Mean Sq**	**F value**	**Pr(>F)**
**Colony**	24	47.72	1.99	1.45	0.17
**Residuals**	30	41.18	1.37		

Journal of Experimental Biology • Supplementary information
